# Care providers’ views on burden of psychosomatic symptoms of IS-traumatized female refugees participating in a Humanitarian Admission Program in Germany: A qualitative analysis

**DOI:** 10.1371/journal.pone.0239969

**Published:** 2020-10-05

**Authors:** Caroline Rometsch, Jana Katharina Denkinger, Martha Engelhardt, Petra Windthorst, Johanna Graf, Christoph Nikendei, Stephan Zipfel, Florian Junne

**Affiliations:** 1 Department of Psychosomatic Medicine and Psychotherapy, University Hospital Tuebingen, Tuebingen, Germany; 2 Department of General Internal Medicine and Psychosomatics, University Hospital Heidelberg, Heidelberg, Germany; Medical University of Vienna, AUSTRIA

## Abstract

**Background:**

Traumatized refugees often suffer from diverse psychosomatic symptoms. Female Yazidi refugees from Northern Iraq who survived attacks of the so-called “Islamic State” were brought to Germany to receive special medical and psychotherapeutic treatment in a unique worldwide humanitarian admission program (HAP). Here, we report on their psychosomatic symptoms and helpful strategies from the perspective of care providers.

**Methods:**

Care providers (N = 84) in this HAP were interviewed in an individual setting as well as in focus groups to gather information about the HAP beneficiaries’ psychosomatic symptoms. Data analysis followed Qualitative Content Analysis by Mayring.

**Results:**

The care providers reported five main psychological burdens of the Yazidis: 1) insecurity regarding loss, 2) worries about family members, 3) ambivalence about staying in Germany or returning to Iraq, 4) life between two worlds and 5) re-actualization of the traumatic experiences. The predominant psychological symptoms the care providers noticed were fear, depressive symptoms, feelings of guilt, and sleep and eating disorders. Regarding somatic symptoms, the care providers mainly received complaints about pain in the head, back, chest and stomach. Helpful strategies for providing adequate health care were care providers’ cooperating with physicians, precise documentation of beneficiaries’ symptoms, and additional support in directing the beneficiaries through the health care system. Regarding psychotherapy, interpreters help to overcome language barriers, onsite psychotherapy, flexible therapy appointments, psychoeducational methods, time for stabilization, and support in coping with daily life aspects. In the care providers’ experience, psychotherapists have to build a relationship of trust. After grief therapy, a trauma-specific therapy in a culturally adapted way is possible.

**Conclusion:**

The HAP is a unique model health care program to offer highly traumatized refugees medical and psychological help. Care providers reported on several (psycho-)somatic symptoms of the traumatized women. The strategies the HAP care providers perceived as helpful can be recommended for similar projects in the future.

## Introduction

According to numbers from the Office of the United Nations High Commissioner for Refugees (UNHCR), in 2019, there were around 25.4 million refugees worldwide [1], of which 6.5 million refugees were from Syria, 2.6 million refugees from Afghanistan and 2.4 million refugees from South Sudan [2]. The majority (85%) of all refugees were living in host countries with low or middle income, e.g. Turkey, with around 3.5 million refugees, Pakistan and Uganda, with around 1.4 million refugees each [2]. Refugees are individuals who were displaced from their country of origin due to their belonging to a particular race, religion, nationality, social group or political opinion [3]. The high numbers of refugees, and their increased vulnerability to suffer from mental health disorders, [4] demonstrate the impact their mental health status has on the public health of each host country [5]. Overall, the prevalence of PTSD in refugees in the first years of resettlement is higher than the PTSD prevalence in the host country. Five years later, the prevalence of depressive and anxiety disorders in refugees is increased compared to the time after resettlement [5]. In refugee samples, there is a prevalence of around 30% for PTSD [6, 7]. However, Iraqi refugees show a prevalence of PTSD of up to 37.2% [8], and studies even show a PTSD prevalence of 42.9% in the Yazidi population [9]. The Yazidis are a religious minority mainly living in the Sinjar area in Northern Iraq [10]. One study investigating these Yazidi women living in Germany shows PTSD prevalence up to 98% [11] and prevalence rates of 39% for anxiety disorder and 19% for depression [12]. In August 2014, the so-called “Islamic State” (IS) attacked several of their villages and forced thousands of Yazidis to convert to Islam; many men were killed, women were kidnapped and fell victim to sexualized violence in large numbers [13–15]. In June 2016, the UN declared these atrocities as an ongoing genocide of the Yazidi people [16].

To ensure medical and psychosocial treatment for the survivors, the federal state of Baden-Wuerttemberg initiated a Humanitarian Admission Program (HAP), which brought 1100 traumatized women and children to Germany. Alongside with health care, the so called Special-Quota Project aimed to integrate the women and children into society by giving them German lessons and integrating the children in school [14]. After such tremendous atrocities, it is common for refugees to suffer from somatic comorbidities alongside mental disorders such as PTSD [17, 18]. Refugees from non-Western countries seem to suffer more often from unexplained somatic symptoms than the general Western population [19]. In particular, chronic pain shows to be a frequently observed symptom [20]. There is evidence that poor social integration, poor social support, poverty and restricted health care access lead to even higher rates of mental disorders among refugees and migrants [5, 21]. However, there still is insufficiency in providing basic needs such as food, water, sanitation and health care for refugees [22] even though access to physical and mental health services is required internationally by law for refugees [23]. Due to the high prevalence of PTSD after a traumatic experience therapies include psychological and pharmacologic interventions [24]. Recommendations support trauma-focused cognitive and exposure-based therapies as first line treatment option [25]. Exposure therapy include the techniques of Prolonged Exposure, cognitive therapy, cognitive processing therapy and eye movement desensitization and reprocessing (EMDR) [26]. Recent research focus on EMDR to answer a possible effectiveness as intervention for patients with chronic pain [27]. Refugees face many access barriers to mental, physical and psychosocial support [18, 28]. A recent systematic review identified language, help-seeking behaviors, lack of awareness, stigma, and negative attitudes towards and by care providers as the main barriers to accessing health services in a refugee context [28]. To provide sufficient health care, there is need for more information about the country’s health care system for refugees and support for health care professionals in providing culturally appropriate treatments [29]. Physicians have to develop an understanding of asylum seekers’ specific health care problems and find a way of appropriate intercultural communication [30]. These difficulties are prevalent across Europe due to policy differences in the country’s particular health care services [31]. However, the German health care system has to provide adequate health care services for refugees, even though there are no procedures or guidelines established in Germany for ensuring a standardized and effective therapy in this specific context [32]. The systematic destruction of the Yazidi community has resulted in health care issues that are extraordinary and arise from subjective and collective aspects and cultural traumatization that require a special concept of integrated medical care [33].

When discussing medical and mental health care in the context of traumatized refugees, it is important to also keep the care providers in mind. There are first results on the impact of working with traumatized populations on care providers. In a study within the Special Quota Project, 23% of participating care providers showed symptoms of secondary traumatization [34]. Generally, care providers, in particular social workers, seem to be highly psychological stressed by their exposure to trauma content [35]. Due to their high involvement in their clients’ life and their professional experience and knowledge in interpreting behavior and symptoms, investigating the care providers’ views on their clients and on mental health care provides give important insights.

Since there is still need for understanding mental health care systems, service delivery and treatment effectiveness [36], this study aims to describe the views and experiences of professionals working with Yazidi refugees to assess the psychosomatic symptoms of this traumatized sample. Furthermore, the professionals’ working experiences were analyzed to offer medical and psychotherapeutic provision and deduce helpful strategies.

## Methods

This paper summarizes somatic and psychological burdens as well as helpful strategies for the health care of traumatized female Yazidis from the care providers’ perspective.

### Study design and ethical considerations

This explorative, cross-sectional study was conducted within a mixed-methods design. Since the quantitative results of this study are already published [37], the present paper illustrates the qualitative data derived from ten focus groups with care providers of the Humanitarian Admission Program (HAP) complemented with aspects from individual interviews. Individual interviews with the care providers were added to gain further information that could have been neglected in the focus groups due to phenomena such as social acceptance and social desirability. The present study was approved by the ethics board (ethics application 189/2017BO2) of the medical faculty at the University and University hospital of Tuebingen; it fulfills the ethical principles of the Declaration of Helsinki [38].

### Study conduction

Psychologists, physicians and scientists of the Department of Psychosomatic Medicine and Psychotherapy and from the Department of Child and Adolescent Psychiatry and Psychotherapy of the University Hospital of Tuebingen moderated the focus groups after they had received a specific training to increase the comparability of the ten focus groups. In the preparatory training, the volunteers received an interview guide covering the main research questions, but also hints for further questions if needed. Furthermore, the interviewers were given background information about the study and the HAP as well as instructions on how to conduct the focus groups in a standardized way. The individual interviews were conducted by three specially trained researchers from the Department of Psychosomatic Medicine and Psychotherapy at the University Hospital Tübingen.

At the beginning of each interview and focus group, participants were informed about the study aims and conditions. Each care provider gave written consent before participating in the study. Interviews and focus groups were audio-recorded and transcribed afterwards, whereby personal data was anonymized in the focus groups and pseudo anonymized in the interviews. The focus groups lasted 68 to 81 minutes, and the complemented individual interviews 14 to 31 minutes.

### Sample description

All registered care providers working in the HAP were informed about the study and invited to participate (N = 132), of which N = 96 participated in the study (response rate = 72.7%). Participants were 96 care providers with different professional backgrounds such as psychotherapists/physicians, social workers, interpreters, creative therapists, administrators, and volunteers working in the HAP. Administrators were excluded from the focus group and from this analysis, for that our study population numbered N = 84. Each focus group consisted of six to ten participants and was homogeneous regarding the respective professions. Five focus groups consisted of social workers. Psychotherapists and psychologists formed two focus groups. One focus group each was held with interpreters, art therapists, and volunteers. Study participation was voluntary and conducted in German language.

### Assessment instruments

Sociodemographic Data and working conditions were assessed using a self-developed questionnaire.

A short semi-structured guideline for the focus groups and individual interviews was developed by the research team using the SPSS-method by Helfferich [39]. Key questions for the focus groups and the single interviews were: 1. “What are the women’s (HAP beneficiaries) heaviest burdens?” and 2. *“*What are the women’s (HAP beneficiaries’) needs, and in which aspects do you support them?” Further, we asked for suggestions to improve the health care system for traumatized refugees like the present sample, in particular concerning psychotherapy, but also regarding other challenges.

### Data analysis

Qualitative content analysis after Mayring [40–42] was conducted using the software MAXQDA [43]. Qualitative content analysis is a technique for analyzing recorded and transcribed communicative material with the aim of a systematic examination [42]. It is used in many areas of psychological, pedagogic and sociological research [40]. For all information of the focus groups this program gives numbers and positions to ensure from where single quotes derive, which we will state in brackets in the results section.

**Fig 1:**
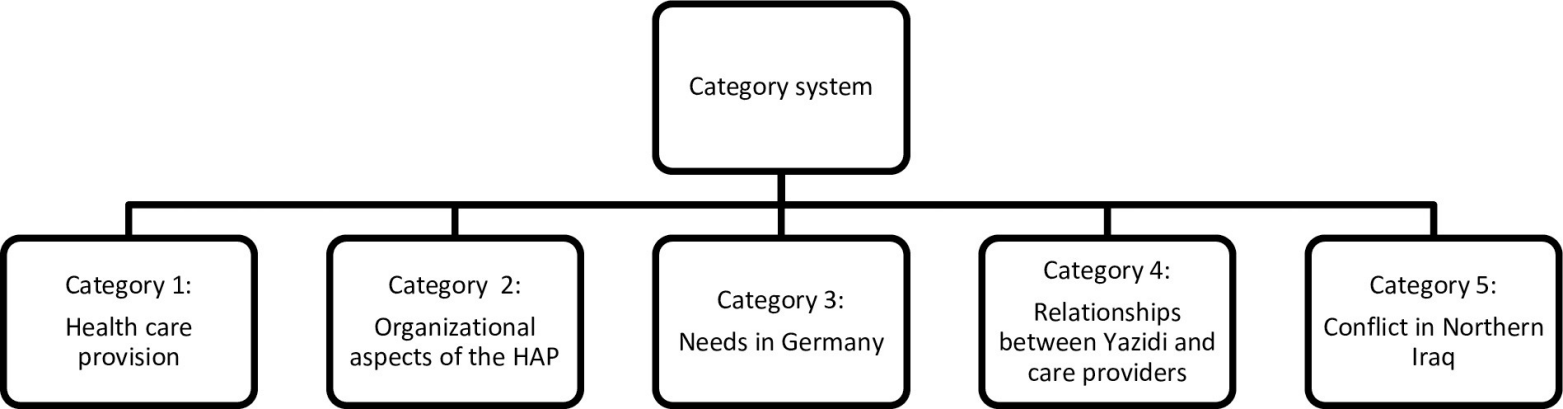
Code book with the main categories of the qualitative data derived from the analyses of the focus groups and single interviews with the care providers in the HAP.

In a first step, inductive categories were derived by a summarizing analysis of a sample of transcripts. In a deductive way, categories derived from the interview guide and recent literature were added. Through structuring and hierarchizing these different categories a codebook was created covering the main topics of the transcripts regarding the research question [41]. Two independent raters evaluated the completeness of the codebook in a test trail. After this revision loop, the research team agreed on a finalized version of the codebook with five main categories (Fig 1). After finalizing the codebook, two independent raters assigned relevant text sections of the focus groups and single interviews to the categories of the codebook. In unclear cases, the whole study team was involved in the decision and coding process.

The results of this paper focus on psychosomatic symptoms derived from subcategories of category 1 “health care provision” and category 5 “conflicts in Northern Iraq”: 1. traumatic experiences and psychological burdens, 2. somatic symptoms, 3. psychological symptoms and emotional states, 4. medical needs and provision, and 5. psychological needs and provision The quotes in this manuscript were translated from German to English.

Statistical analyses were done by using SPSS (version 24.0.0.1 [44]). Sociodemographic variables of the sample were analyzed using the mean (*M*) and standard deviation (*SD*).

## Results

### Sociodemographic variables

Care providers were mainly females (90.4%), with a mean age of 44.57 years (*SD* = 13.00). More than half of the care providers (64.3%) reported being social workers. At the time of data collection, the care providers had worked for 15.87 months (*SD* = 6.59) in the HAP, with a mean of 18.1 (*SD* = 13.99) working hours per week. Each care provider said to be responsible for a mean of 18.63 beneficiaries (*SD* = 25.98), some care providers were responsible for children in first line and not for the women, despite we included these care providers due to their experiences of the women’ symptoms by their work with their children to gain further information. Some of the care providers (46.3%) had special training for treating traumatized refugees. Table 1 provides more information regarding the sample description.

**Table 1:** Sociodemographic data and working conditions of the care providers in the HAP.

Sample description			
Age (years)			
	Mean	44.57	
	SD	13.00	
	Range	23–66	
Gender			
	Female	75	90.4%
	Male	8	9.6%
Profession			
	Social education worker/social worker	54	64.3%
	Interpreter	11	13.1%
	Psychologist/psychotherapist	6	7.1%
	Creative/special therapist	6	7.1%
	Psychotherapist for children and youth	3	3.6%
	Medical practitioner/psychiatrist	1	1.2%
	Other profession	3	3.6%
Additional training in the treatment of traumatized refugees			
	Yes	37	46.3%
	No	43	53.8%
Period of time in the project (months)			
	Mean	15.87	
	SD	6.59	
	Range	1–26	
Number of women cared for			
	Mean	18.63	
	SD	25.98	
	Range	0–111	
Working time per week (hours)			
	Mean	18.1	
	SD	13.99	
	Range	1–48	
Direct contact with Yazidis (hours)			
	Mean	12.11	
	SD	10.90	
	Range	0–39	

### Traumatic experiences and psychological burdens

**Fig 2:**
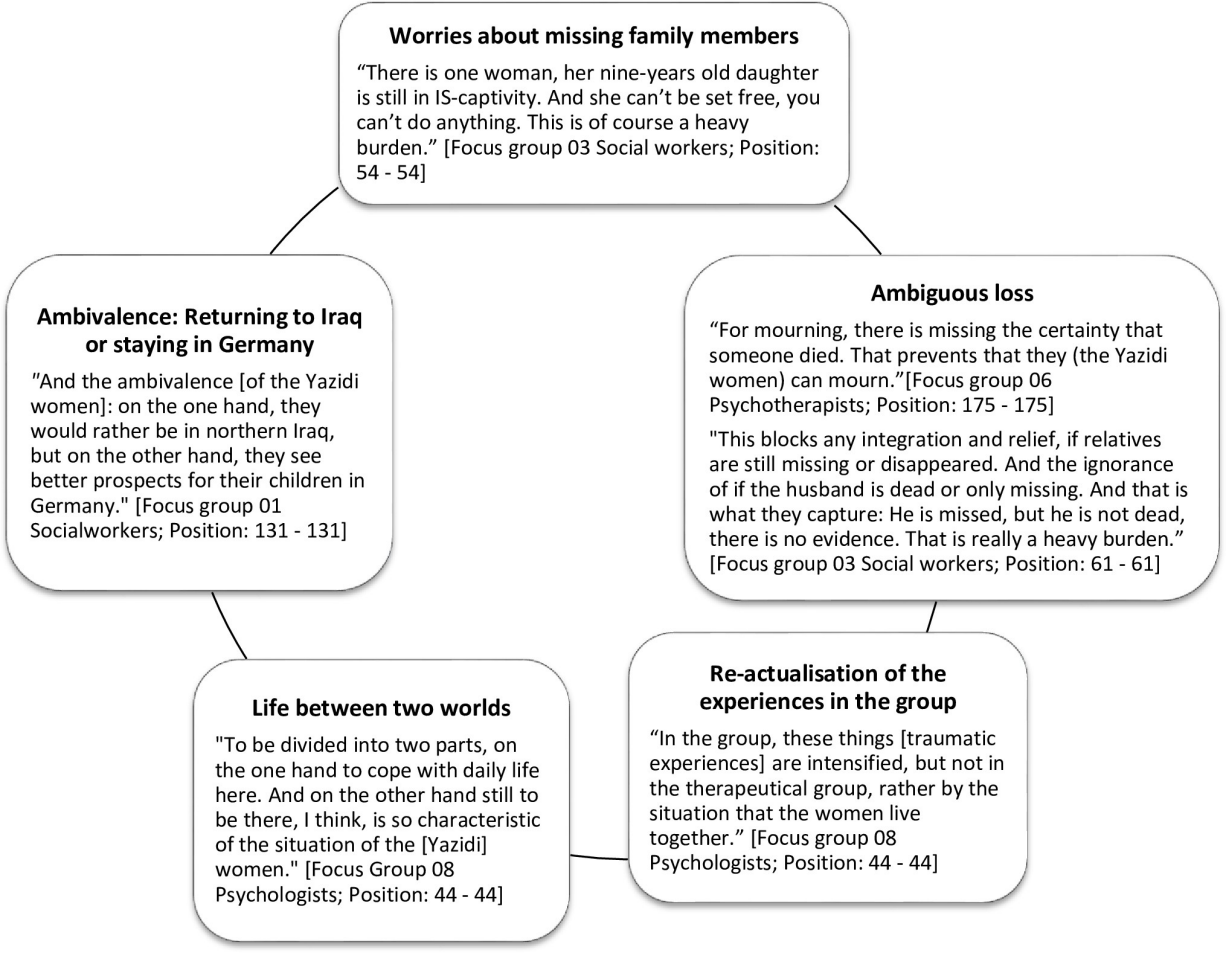
Main psychological burdens of female Yazidi refugees by the perspective of the care providers.

The following paragraph contents data of category 1 (“health care provision”) from the above-mentioned codebook (see Fig 1). During the focus group sessions, the care providers explained that the HAP beneficiaries suffer tremendously as a result of their traumatic experiences. Each beneficiary suffered different traumatic experiences described by one social worker as an “individual scenario” [Focus group 01 Social workers; Position: 34–35]. These experiences are based on a “re-traumatization” what is further considered as re-acutalisation [Focus group 06 Psychotherapists; Position: 52–52] and “loss-trauma” as mentioned by a psychotherapist [Focus group 06 Psychotherapists; Position: 52–52] to describe the genocides in the past of the Yazidi community. These involved different emotional and psychological states and processes, such as feelings of missing family members, sorrow, history of war, and sorrow of earlier losses due to conflicts in a community-focused culture. The following figure (Fig 2) describes the main and most common psychological burdens the HAP-beneficiaries express to their care providers. The figure demonstrates that all symptoms were experienced by the care providers, the relevance of them can be considered as equal.

### Somatic and psychological symptoms

In this section, we present the results of the care providers’ reports on the HAP-beneficiaries’ somatic and psychological symptoms derived from category 1 (“*health care provision*”) of the above-mentioned codebook.

### Somatic symptoms

From the care providers’ reporting, pain is the dominant somatic symptom of the traumatized Yazidi women. This finding is consistent, independent of the care providers’ professional backgrounds. Upon the moderator’s enquiry, the care providers specified the pain symptoms to the following body locations: “headaches, pain in the chest, stomach, abdomen as well as back aches” [Focus group 01 Social workers; Position: 58–63]. For an overview of the main symptoms see Table 2.

**Table 2:** Somatic symptoms of the traumatized Yazidi women from the care providers’ perspective.

Symptoms	Quote
Pain	“It is the permanent topic: pain, those body memories.” [Focus group 02 Social workers; Position: 633–633]
Psychosomatic symptoms	“Of course, some women show psychosomatic symptoms such as sleep disorders or eating disorders.” [Focus group 02 Social workers; Position: 631–631]
Fainting and seizures	“The main need in the beginning was simply stabilization; many women had seizures constantly and were fainting.” [Focus group 09 Art therapists; Position: 40–40]
Sleeping disorders	"A 19-year-old who has very strong sleep disorders said: I can only sleep when my mother is there. The [sense of] security is missing.”[Focus group 06 Psychotherapists; Position: 183–183]
Somatization	“I realized a massive physical burden in the women […] a physical burden, that is connected with a psychological burden” [Focus group 10 Volunteers; Position: 21–21]

### Psychological symptoms and emotional states

Regarding psychological symptoms and the beneficiaries’ emotional states, our analyses resulted in nine main symptoms: feelings of guilt, depression, social withdrawal, rumination, sleep problems, nightmares, hopelessness, anger, fear and fainting (for citations, see Table 3). The beneficiaries express these symptoms as nightmares, mental tension and sleep disorders. With high priority, the care providers reported depressive symptoms, the Yazidis “are not allowed to be happy. They have a guilty conscience; if they feel happiness, they immediately have to suppress it” [Focus group 03 Social workers; Position: 58–58]. One psychotherapist concluded that the “trauma process […] results in massive depression” [Focus group 06 Psychotherapists; Position: 21–21]. She explained an obstruction of this healing process due to the unknown residence of the IS-fighters. The psychological constitutions of some Yazidi women can be revealed in their idea “to belong to the IS. To all [Yazidis] was said [by the IS] that they belong to them, their whole life.” [Focus group 06 Psychotherapists; Position: 102–103] More information is summarized in Table 3.

**Table 3:** Descriptions of psychological symptoms and emotional states of traumatized female refugees reported by care providers.

Psychological symptoms and emotional states	Quote
Feeling of guilt	"Yes, they cannot be happy, they feel guilty when they feel joy, and they have to suppress the feeling of joy right away."[Focus group 03 Social Workers; Position: 58–58]
	“The mothers have clearly stated that they would rather be dead because their siblings or other family members have died. And that they just think they have no right to live anymore.” [Focus group 09 Art Therapists; Position: 42–42]
Depression	"Primarily, they come in because of depressive symptoms, […] tensions and sleep disorders.” [Focus Group 06 Psychotherapists; Position: 162–162]
Social withdrawal	"Then she sits alone all day in the room and hears very sad music. She is withdrawing from others to her room." [Focus group 05 Social workers; Position: 78–78]
Rumination and Sleeping problems	"The worries about the families that one always thinks about the situation at home or the things that happened, that they cannot sleep at night. Those factors have consequences on the day." [Focus group 10 Volunteers; Position: 32–32]
Nightmares	"And what we experience in everyday life is [that the women ask] ‘We have nightmares, what should we do? I always see the IS fighter in front of me.’”[Focus Group 01 Social Workers; Position: 47–47]
Hopelessness	“[One woman asked] ‘For whom? It's not worth it anymore, it's no longer worth it for me to be able to enjoy life or somehow to feel happy again, for what, for whom?’”[Focus group 03 Social workers; Position: 59–59]
Anger	And there is a lot of anger! A lot of anger! I think the anger is immense. They [the Yazidi women] are so angry at their fate that this happened to them. And I think everything else is worth nothing [compared to the suffered harm]. Their whole life has been ruined.” [Focus group 09 Art Therapists; Position: 39–39]
Fear	“It is the fear that another man […] creeps around the house. The doors are often closed.” [Focus group 01 Social workers; Position: 48–48]
Fainting	“In the beginning, after their arrival, at least ten women kept fainting and had very heavy fears at night.” [Focus group 09 Art Therapists; Position: 154–154]

### Health care system and needs

In these paragraphs, the care providers’ evaluations of the health care system and the health care needs of the HAP beneficiaries are presented.

### Medical needs and provision

From the care providers’ perspective, the following four aspects should be considered as best practice in refugee health care:

- Cooperation between physicians with different specializations to ensure close contact and follow-up examinations
- Documentation of the beneficiaries’ symptoms by the care providers or the beneficiaries themselves for the physician to reduce language barriers
- Care providers and physicians should document the medical care and check-ups to ensure security for the beneficiaries, e. g. to avoid the feeling of the Yazidi women to be misunderstood, further documentation can help to determine if an interpreter is necessary to support the doctor’s rounds
- Care providers should support the beneficiaries in making a decision about how urgently medical support needs to be received or if it is possible to wait and watch the symptoms for some days

Regarding the frequency of medical support, the care providers had differing experiences. On the one hand, over-utilization of the medical health care system, and on the other hand, refusal of medical examinations as a social worker reported, “There are many conflicts, because they [Yazidi women] refuse to go to the dentist whether they have toothache or not.” [Focus group 01 Social worker; Position: 180–180].

Difficulties also occurred regarding the acceptance and compliance with medications. Many beneficiaries do not want to take any medications because, as a social worker reported, “they were sedated and immobilized with pills [in IS-captivity] because they were kept all together in one room” [Focus group 03 Social worker; Position: 123–125]. Further reasons for refusing medication result from gaining weight or tiredness as side effects of psychopharmaceutic. However, there seems to exist more acceptance of taking medications in other accommodations, as another social worker describes even a “craving” for medication, regardless which category of medication.

### Psychological needs and provision/access and acceptance of psychotherapy

At the date of data collection, the HAP had already existed for 1.5 years, during which the care providers developed several experiences to establish structures of psychological therapies for the beneficiaries. On the one side, the care providers pointed out the need for simple and direct access to the provision of psychological services in the beneficiaries’ accommodations, and on the other side, there are recommendations for psychotherapists to ensure acceptance and support of the psychotherapy. The detailed helpful strategies are presented in Table 4.

**Table 4:** Helpful strategies to improve access to and acceptance of psychotherapy for traumatized female refugees.

Access to the provision of psychotherapy	Acceptance of psychotherapy
• Availability of interpreters	• Explanation of mental disorders and mental symptoms
• Offer of low-threshold access and offers for building relationships with therapists	
	• Psychoeducational groups as easier access to psychological therapies
	• Explanation of differences between psychotherapy and medications
• Understanding the needs and acceptance of the Yazidi women	
	• Consider cultural differences
	• Giving time at the beginning for coping with everyday situations
• Providing on-site psychotherapy in the accommodations	
	• Time to develop acceptance of the use of psychotherapy
• Psychiatrists’ rounds on-location	
	• Ensure sufficient number of therapy hours
	• Ensure gaps in daily schedules for going to a psychotherapist
• Supported psychological therapy by offering company overall for older Yazidi women	
	• On-site psychotherapy
	• Offer open therapy instead of fixed appointments
• Clarify medical diagnosis for any therapy	
	• Accept the first step of sorrow and grief before making trauma-specific therapy
• Ensure individual therapy with support of interpreters	
• Ensure financial support of trauma-specific psychotherapy long after arrival in the host country	

Regarding psychological individual and group therapies, the care providers emphasized the importance of time for both listening to the beneficiaries’ needs and accepting that they are not yet in the stage to go through their atrocities e.g. in trauma exposure therapy. In particular, for group psychotherapy, the study shows the need for organizational aspects such as the availability of interpreters and locations for psychotherapy, as well as the need for psychoeducation that was in particular mentioned by the care providers for the group psychotherapy. Further results can be seen in Table 5.

**Table 5:** Helpful strategies for individual and group psychotherapy from the care providers’ perspectives.

Aspects of individual psychotherapy	Aspects of group psychotherapy
• Time to listen to the Yazidi women	• Find adequate intervals for therapy
• Accept when the women do not want to share traumatic experiences	• Organization of room for therapy
	• Availability of interpreter
	• Psychoeducation
• Offer therapeutic strategies to speak of “we” or other women	• Behavioral and psychodynamic psychotherapy
	• Building relationships and finding trust in each other

### Further (therapeutic) offers by the different care providers

The care providers reported several help offers and strategies they considered supportive for the HAP beneficiaries:

- Art therapy
- Basic conversations
- Open groups with different topics and information (e. g., society, medicine)
- Support in coping with everyday life (food, living, clothes, hygiene, school problems, shopping, household)
- Taking their minds off things (sports, football, handball, dancing, Zumba, swimming, running, exhibition, cooking, baking, handicrafts, spinning wool, knitting, sewing, felting, organize and participate in celebrations, helping in garden works)

## Discussion

In this study, care providers of a German humanitarian admission program reported several psychosomatic symptoms of highly traumatized female Yazidi refugees. The care providers considered the following psychological burdens as predominant in their clients: ambiguous loss, ambivalence of returning to Iraq or staying in Germany, worries about family members, and the re-actualization of the traumatic experiences. Psychological symptoms by the care providers’ perspective are fear, depressive symptoms, sleep disorders, and eating disorders. Regarding somatic symptoms, the care providers mainly described pain symptoms in the traumatized women such as headaches and pain in the chest, stomach, abdomen, and back. Furthermore, the qualitative analysis resulted in several helpful strategies for providing health care offers: care providers’ cooperation with physicians, precise documentation of the beneficiaries’ symptoms as well as support to guide the traumatized women in the health care system to meet the medical needs. For offering psychotherapy, there are challenges to ensure access and acceptance, such as the availability of interpreters, on-site psychotherapy, flexible therapy appointments, psychoeducational methods as well as leaving sufficient time for stabilization and regaining a daily life. The results of this study demonstrate the importance of building a relationship of trust between psychotherapists and refugees. Further therapy offers, such as art therapy and support in daily issues, proved to be promising.

The qualitative analysis of the psychological burdens and symptoms from the care providers’ perspective is in line with the quantitative survey results showing that in the care providers’ view, worries about the family, as well as nightmares, insomnia and depression are the highest stressors for the IS-traumatized women [37]. The fact that many refugees suffer from sorrow or even from a prolonged grief disorder (PGD) [45] and worries about their relatives [46] can be understood based on ongoing conflicts in many home countries, and in the specific case of the Yazidis, ongoing violence against family and community members since many are still missing or in IS captivity [47]. With a focus on the worries about the family, a recent study on Iraqi refugees shows that these emotional stressors are a significant risk factor for PTSD [48]. Further, fear after traumatic experiences can be understood as part of the PTSD itself [49]. The literature shows that the diagnosis of anxiety disorders can be found in refugees with a worldwide origin, with a prevalence of up to 50% [50], and in refugee sample grading for asylum in Europe, the prevalence rate is described as up to 31% [51]. However, a comorbidity between PTSD and anxiety disorders should also be considered [52]. We found pain symptoms in recent investigations as the heaviest somatic burden and symptom in the Yazidi sample [37, 53]. Low back pain symptoms are also described in other refugee samples [54]. The results of this study underline further the role of pain in traumatized refugees and the need for evidence for treatment options [20].

Helpful strategies for the health care system of this study have to be taken into account due to the fact that Iraqi refugees are resettled worldwide with high rates of PTSD [8], and more than half of the refugees report at least one traumatic experience [55]. The urgent need for adequate health care provision with simplified access to the health care system and treatment options as well as guidelines for the health care system are discussed intensively in the arrival countries [56–58] but also in Germany [59]. Interestingly, there is a moderate interest in the research of mental and medical health care of refugees and, in particular, most in chronic diseases in the last 25 years [60]. Several studies highlight the following aspects as good practice for refugee mental health care: a high relevance of culture-specific aspects and intercultural training of health professionals [61], use of a transcultural approach for assessment and screening and trauma-informed health care [62], implementation of cultural mediators [57], social integration, providing information about available services [63], conducing of screening by using further screening instruments after the refugees’ arrival [64, 65], faster asylum procedures, social interactions with the surrounding society [66], need for interpreters [67], and the consideration of individualistic vs. collectivistic cultures [67].

Nevertheless, there is only little evidence of more detailed needs and skills in offering health care services, as investigated in the present study. Generally, this study’s findings are in line with a World Health Organization publication from 1996, in which helpful skills and suggestions are provided for treating refugees who are survivors of torture, violence and rape [68]. In our study the care providers shared essential experiences for offering medical and psychological support for IS-traumatized females. A close cooperation between physicians and providers in general was also found in health care analysis in the United States for successful care coordination, as well as direct scheduling of appointments and case management by care providers of refugees [69]. They also identified the importance of trust in relationships among providers and refugees [69]. There is a high need for basic trauma therapy for refugees, as shown in a sample of Syrian refugees [70]. For that, psychological interventions are investigated more and more in the literature. There is evidence of trauma-focused psychological interventions (such as EMDR and narrative exposure therapy) for refugees with a PTSD diagnosis, as shown in a recent review and meta-analysis [71]. Studies on Iraqi refugees underline this evidence [72]. We found that on-location treatment offers a successful strategy for the HAP beneficiaries in this study. This concept is discussed in a refugee context as a home based primary care in the United States with, until now, missing evidence due to the lack of research [73]. Further, studies show that stepped care delivery with consideration of socioeconomic and cultural influences as well as disorder-specific therapies are important for the health care system of refugees [36]. For that, there are new approaches in offering health care services, such as the HAP we investigated in this study. To integrate the psychotherapeutic provision in the health care system, a study course was founded in Northern Iraq to educate psychotherapists to be on-location at the camps of the Yazidi population [74]. Another way is a multidisciplinary treatment approach for traumatized refugees in specialized treatment centers that include psychotherapy, medical and social interventions [75]. Generally, the HAP was constructed in a way to assess the main (psycho-) somatic symptoms as experienced by the care providers in this study by offering psychosocial, medical and psychosomatic therapies for each accommodation. Finally, next to these reported projects for health care services for refugees, there is a, urgent requirement for political solutions to guarantee universal health coverage [22]. Politicians of all nations have to focus their attention on the challenges through the high number of refugees and adopt each health care system for the special demands [22].

The HAP seems to be a successful politically initiated health care model for traumatized refugees and can be seen as a learning opportunity to improve provision of mental health services for refugees in a high-income country.

## Limitations

Using a qualitative design and the care providers’ perspective, there can be biases due to subjectivity and over- or underestimation of the somatic and psychological symptoms. Well known is an underestimation of psychological disorders such as PTSD in the general population [76] what has to be analyzed separately for each (psycho-) somatic symptom as well as for every profession of the HAP in further research. The results of this study are based on the insights of the professionals who work with the traumatized refugees and not on the opinions and experiences of the Yazidi women themselves. Though the care providers are in close contact with the beneficiaries, we see them as a valid group for investigation. Our analysis is not able to give quantitative analyzed information on the different experiences of the included care providers’ profession. Further research should focus on the different care providers’ profession to gain results of their experiences. For the second research question of this study, the analysis of helpful health care strategies, the care providers’ experiences are essential for summarizing needs for health care provision of an extremely traumatized refugee sample. The HAP is a special and unique program where the traumatized refugees were brought actively to Germany to receive medical, psychosocial and psychotherapeutic services and support in an organized and highly dosed way. This must be considered for generalization of the study’s results. Selection biases can occur because participation was voluntary and more interested and engaged care providers could have taken part in this study. In further research there should be focus on the differences between the regions where the traumatized women are accommodated but also a follow-up is from high interest. Clearly, the traumatized women have to be investigated in a one-to-one interview setting, as well as other countries in which similar projects are planed to compare our study results.

## Conclusion

Care providers reported several somatic and psychological symptoms in IS-traumatized female refugees. Mainly, they report about pain in the head, back, chest and stomach as well as fear symptoms, depression and nightmares. The HAP is a unique model of psychosocial care for traumatized female refugees to ensure, e.g. medical and psychological support. Experiences reported here may be a useful resource for similar admission programs and specialized care for this very vulnerable group of beneficiaries.
